# Microarray profile of circular RNAs identifies CBT15_circR_28491 and T helper cells as new regulators for deep vein thrombosis

**DOI:** 10.3389/fcvm.2025.1578711

**Published:** 2025-06-30

**Authors:** Weiwei Chen, Ying Zhu, Sihua Niu, Yan Zhou, Jian Chang, Shujie Gan

**Affiliations:** ^1^ Department of Nursing, Shanghai General Hospital, Shanghai Jiao Tong University School of Nursing, Shanghai, China; ^2^ Department of Clinical Pharmacy, Shanghai General Hospital, Shanghai Jiao Tong University School of Medicine, Shanghai, China; ^3^ Department of Vascular Surgery, Shanghai General Hospital, Shanghai Jiao Tong University School of Medicine, Shanghai, China

**Keywords:** deep vein thrombosis, circular RNAs, circRNA–miRNA–hub gene network, immune cells, hub genes

## Abstract

**Background:**

Deep vein thrombosis (DVT) is the third most common cardiovascular disorder and can lead to high mortality and morbidity. This study aimed to clarify the molecular and immune characteristics of circular RNAs (circRNAs) and messenger RNAs (mRNAs) in DVT progression.

**Methods:**

DVT-associated dataset GSE148333 was downloaded to screen differentially expressed circRNAs and mRNAs using the limma package. DVT-related modules/genes were then filtered using WGCNA. Subsequently, key hub genes associated with DVT were determined using four algorithms: MCC, MNC, EPC, and DEGREE. A circRNA–miRNA–hub gene network was then constructed, and the relationship between the DVT-related hub genes and immunity was analyzed. Finally, a DVT rat model was established to verify the expression of critical circRNAs and hub genes using real-time quantitative PCR.

**Results:**

A total of 421 circRNAs and 1,082 mRNAs were differentially expressed in DVT. Among these, 235 circRNAs and 207 mRNAs were identified as DVT-related and were significantly enriched in signaling pathways including NOD-like receptor, mTOR, FoxO, p53, and cell cycle. Thereafter, 17 important hub genes were obtained, including *Birc5*, *Plk4*, *Dlgap5*, *Spag5*, *Cdca2*, *Ccnb1*, *Cdca8*, *Kif18a*, *Kif2c*, *Espl1*, *Cenpu*, *Cdc20*, *Ncapg*, *Asf1b*, *Nek2*, *Aurkb*, and *Cenpw*. Subsequently, 227 circRNA–miRNA pairs and 84 miRNA–hub gene pairs were included to construct a circRNA–miRNA–hub gene network, containing CBT15_circR_28491-rno-miR-139-3p-*Kif18a*/*Cdca8*/*Nek2*. Eight immune cell types showed differential infiltration levels in DVT and controls, with T helper cells positively related with all 17 hub genes.

**Conclusions:**

This study offers valuable information about circRNAs and mRNAs in DVT, identifying CBT15_circR_28491-rno-miR-139-3p-*Kif18a*/*Cdca8*/*Nek2* as a potential target for DVT management.

## 1 Introduction

Lumen stenosis and venous return obstruction are signs of deep vein thrombosis (DVT), resulting from abnormal coagulation of blood in the deep veins (1). According to statistical data, 90% of venous thromboembolisms (VTEs) are caused by DVT, which is accepted as the third most common cardiovascular disorder. Clinically, 90% of VTEs are caused by DVT (2). Approximately half of DVT cases are linked to transient risk factors, and these events are often preventable with prophylaxis, including direct oral anticoagulants and vitamin K antagonists. However, once VTE is diagnosed, many patients require long-term anticoagulation. Meanwhile, 25%–40% of DVT patients experience post-thrombotic syndrome, which significantly impacts their quality of life (3). Further comprehensive research is needed to improve the prevention and early diagnosis of DVT.

Several risk factors have been reported to contribute to the development of DVT. In particular, with the advancement of high-throughput sequencing, more genomic information has been captured, which was superior to traditional population-based risk algorithms for identifying patients with different diagnoses, prognoses, and responses to specific drug treatments (4, 5). Although the molecular mechanisms underlying DVT remain unclear, previous evidence suggests that non-coding RNAs may be involved in its development (6). Circular RNAs (circRNAs), a class of non-coding RNAs, can regulate gene expression by acting as microRNA (miRNA) sponges. In recent years, several studies have reported the roles of circRNAs in the diagnosis and treatment of cardiovascular disease (7, 8). For example, hsa_circ_000455 has been identified as an important biomarker in the pathogenesis of DVT by sponging hsa-miR-22-3p and subsequently targeting NLRP3 (6). Similarly, Lou et al. showed that the hsa_circ_0001020/miR-29c-3p/MDM2 axis may serve as a prospective therapeutic biomarker for DVT (9). Despite significant progress in identifying molecular biomarkers, further investigation into high-risk dysregulations is still needed to improve DVT management.

In addition, we focused on identifying hub genes—key regulatory molecules that occupy central positions in gene co-expression or protein–protein interaction (PPI) networks—in our bioinformatics analysis. Hub genes are characterized by high connectivity (degree), interacting with numerous other genes or proteins, and often play crucial roles in modulating biological pathways (10). In disease studies, hub genes are frequently implicated as drivers of pathological processes, making them potential diagnostic markers or therapeutic targets (11–13). For example, in cardiovascular diseases, hub genes such as *F3* (coagulation factor III) and *SERPINE1* (plasminogen activator inhibitor-1) have been linked to thrombotic risk through their central roles in hemostasis networks (14). In this study, we applied weighted gene co-expression network analysis (WGCNA) and degree-based ranking to pinpoint hub circRNAs/messenger RNAs (mRNAs) associated with DVT.

To achieve this, we downloaded GSE148333 to screen for differentially expressed circRNAs and mRNAs between DVT and control samples. The key DVT-related hub genes were then identified by combining results from four algorithms, and a circRNA–miRNA–hub gene regulatory network was constructed. Furthermore, we analyzed the correlation between the expression levels of hub genes and the infiltration of immune cells that showed significant differences in DVT. Finally, a DVT rat model was established to validate the expression levels of crucial circRNAs and hub genes using real-time quantitative PCR (RT-qPCR). Our findings will advance the mechanistic understanding of DVT pathogenesis and identify promising molecular targets for the development of innovative therapeutic interventions.

## 2 Materials and methods

### 2.1 Data source and pre-processing

To obtain the high-throughput expression profile of DVT, we searched the National Center for Biotechnology Information (NCBI) gene expression omnibus (GEO) database (http://www.ncbi.nlm.nih.gov/geo/) using “Deep Venous Thrombosis” as the keyword on 11 May 2024. Because our analysis required the simultaneous detection of circRNAs and mRNAs, only dataset GSE148333 (15) was selected for further analysis. This dataset includes both circRNA and mRNA expression levels from blood samples of three control rats and 12 DVT rats, sequenced using the Illumina HiSeq 2000 (Rattus norvegicus). The expression profile was then standardized using the preprocessCore package (version 19, https://www.bioconductor.org/packages/release/bioc/html/preprocessCore.html) (16) in R version 4.3.1, based on the quantile method.

### 2.2 Screening of differentially expressed circRNAs and mRNAs

Differentially expressed circRNAs and mRNAs between DVT and control samples were screened using the limma package in R version 4.3.1 (version 3.34.7, https://bioconductor.org/packages/release/bioc/html/limma.html) (17), after standardization. A significance threshold of *P* < 0.05 and |log_2_fold change|>1 was applied. The differentially expressed circRNAs and mRNAs were then clustered based on expression levels using the pheatmap package in R (version 1.0.8, https://cran.r-project.org/package=pheatmap) (18), and corresponding heatmaps were generated.

The Database for Annotation, Visualization, and Integrated Discovery (DAVID, version 6.8, https://david.ncifcrf.gov/) (19, 20) was used for functional annotation. To explore the functions enriched among the differentially expressed circRNAs and mRNAs, Gene Ontology (GO) and Kyoto Encyclopedia of Genes and Genomes (KEGG) pathway enrichment analyses were conducted using DAVID. GO analysis included three categories: molecular function (MF). which describes activities at the molecular level; cellular component (CC), which refers to a location; and biological process (BP), which represents molecular activities or processes. *P* < 0.05 was set to determine significant enrichment.

### 2.3 Screening of DVT-related genes based on the WGCNA algorithm

WGCNA is a bioinformatics algorithm used to build co-expression networks, identify disease-associated modules, and screen for important pathogenic mechanisms or potential therapeutic targets. Modules associated with disease states were identified using the WGCNA package (version 1.61) (21) in R version 4.3.1, based on circRNA and mRNA expression levels. The module partitioning thresholds were set as follows: each module contained at least 200 genes, with a cutHeight of 0.995. Modules showing an absolute correlation with disease status greater than 0.3 were retained, and the genes within these modules were considered candidate disease-related genes. Next, overlapping circRNAs and mRNAs between the retained disease-related modules and the differentially expressed circRNAs and mRNAs identified in DVT were defined as DVT-related genes. The cor function in R version 4.3.1 was then used to calculate the correlation between the expression levels of DVT-related circRNAs and mRNAs. Correlation pairs with *P* < 0.05 and an absolute correlation value greater than 0.3 were retained. Finally, a co-expression network of DVT-related circRNAs and mRNAs was constructed and visualized using Cytoscape (version 3.9.0, http://www.cytoscape.org/) (22).

### 2.4 Construction of the PPI network and identification of hub genes

The STRING database (version: 11.0, http://string-db.org/) was used to identify interactions among the protein products of the DVT-related genes (23), and the resulting PPI network was visualized using Cytoscape (version 3.9.0) (22). The constructed PPI network was then analyzed using cytoHubba (version 0.1) (24), a module detection plug-in for Cytoscape3.9.0. Hub genes were identified based on four topological analysis algorithms: Matthews correlation coefficient (MCC), maximum neighborhood component (MNC), edge percolated component (EPC), and DEGREE. After comparing the obtained hub genes by the four respective algorithms, the overlapping ones were defined as important hub genes related to DVT. Furthermore, a principal component analysis (PCA) (25) was performed using the psych package (version 1.7.8, https://cran.r-project.org/web/packages/psych/index.html) in R version 4.3.1, based on the expression levels of the selected important DVT-related hub genes.

### 2.5 Construction of the regulatory network based on the key DVT-related hub genes

First, the sequences of DVT-related circRNAs were extracted, and rat miRNA sequences were downloaded from the miRBase database (https://www.mirbase.org/) (26). The potential interactions between circRNAs and miRNAs were then predicted using the miRanda tool (http://cbio.mskcc.org/miRNA2003/miranda.html), with the following alignment parameters: gap extend = 0, score threshold = 80, energy threshold = −20, and matched sequence percentage threshold = 80% (27).

Next, the target genes of the identified miRNAs were predicted using the miRWalk 3.0 database (http://129.206.7.150/) (28). These were compared with the previously identified DVT-related hub genes, and the overlapping genes were defined as regulatory hub genes (29). Finally, a circRNA–miRNA–hub gene regulatory network was constructed based on the circRNA–miRNA and miRNA–hub gene interactions and was visualized using Cytoscape (version 3.9.0) (22).

### 2.6 Correlation analysis of the key DVT-related hub genes and immunity

Using the single-sample gene set enrichment analysis algorithm in R version 4.3.1, immune cell infiltration in the TCGA dataset was analyzed with the Gene Set Variation Analysis for microarray and RNA-Seq data (GSVA, version 1.36.3, http://www.bioconductor.org/packages/release/bioc/html/GSVA.html) (30). The correlations between immune cell types across samples were then calculated. Later, the Kruskal–Wallis test in R version 4.3.1 was used to compare differences in immune cell infiltration between the DVT and control groups. Finally, the correlation between the expression levels of important hub genes and the infiltration levels of immune cells showing significant differences in DVT was assessed.

### 2.7 Experimental validation of expression levels of the identified crucial circRNAs and mRNAs

Four key circRNAs (CBT15_circR_28491, CBT15_circR_6215, CBT15_circR_10888, and CBT15_circR_40191) and four important hub genes (*Kif18a*, *Cdca8*, *Nek2*, and *Ncapg*) were randomly selected for RT-qPCR in a DVT rat model.

A total of 12 male Sprague Dawley rats (10–12 weeks old, 250–300 g) were purchased from SLAC (Shanghai, China) and housed under controlled conditions: a 12 h light/dark cycle, mean ambient temperature of 23 ± 3°C, relative humidity of 55%–60%, and *ad libitum* access to food and water. After 7 days of acclimatization, the rats were randomly divided into sham and DVT groups (*n* = 6 per group). The DVT rat model was established by inferior vena cava (IVC) stenosis (31). Rats were anesthetized by intraperitoneal injection of 0.3% sodium pentobarbital (30 mg/kg) and placed in a supine position. After a midline laparotomy, the intestines were exteriorized and placed to the animal's left side, and the IVC was carefully isolated from surrounding tissues and partially ligated below the renal veins, with all visible tributaries fully ligated. Rats in the sham group underwent the same surgical procedure without vascular ligation. Postoperatively, all rats were housed under the same environmental conditions with free access to food and water. At 24 h postoperatively, all rats were deeply anesthetized with 2% isoflurane and then euthanized by cervical dislocation. However, before sacrifice, approximately 2 ml of venous blood were collected for subsequent RT-qPCR analysis from the suprarenal IVC under anesthesia induced in a 3%–4% isoflurane chamber and maintained at 1%–2% via face mask. All animal procedures were approved by the Animal Care and Ethics Committee of Shanghai General Hospital (approval no. 2025AW015).

Total RNA was isolated from the blood samples of both groups using TRIzol (Invitrogen, USA), and reverse transcribed into cDNA using the PrimeScript™ RT Master Mix (TAKARA, Japan). RT-qPCR was then performed using SYBR Green PCR Master Mix (Thermo Scientific, USA) on an ABI Viia7 real-time PCR system (Applied Biosystems, CA, USA). The relative expression levels of the circRNAs (CBT15_circR_28491, CBT15_circR_6215, CBT15_circR_10888, and CBT15_circR_40191) and hub genes (*Kif18a*, *Cdca8*, *Nek2*, and *Ncapg*) were calculated using the 2^−ΔΔCt^ method, normalized to internal *GAPDH*. The primer sequences used are listed in Table 1.

**Table 1:** The sequences of all primers.

Primer	Forward sequences (5′−3′)	Reverse sequences (5′−3′)
CBT15_circR_28491	GGTATTCTTTGGTTCTTGCCTCG	ATCCTCCTGCCTCAGCCTCC
CBT15_circR_6215	GCAAGACCCAAAGCCAAGCA	CAAGGGCATCATCCCAGCAT
CBT15_circR_10888	CCCTGGAAATCTGGACACTCT	GGTCTGGGCTGACTTCTGGT
CBT15_circR_40191	TCGCATACATGATGTAATTG	GTGCTTGAGAAGAAGAGGAA
Kif18a	ATCTTACCACTGGCTCTTCT	GACACGGACAACTACTTTCA
Cdca8	AAGCAAATTGAGTCCGACAG	TTTATGGCTTCATCCACCTC
Nek2	TTCCTGGACAGCAAGCACAA	TTCAAGCCATCAGAGTAGCG
Ncapg	TCCGTTCTCCATTCCTTATT	CTTCACCTTCATCTCCCTTT
GAPDH	AGACAGCCGCATCTTCTTGT	CTTGCCGTGGGTAGAGTCAT

### 2.8 Statistical analysis

Data are presented as mean ± standard deviation, and statistical analyses were performed using GraphPad Prism version 9.0. Comparisons between the two groups were conducted using an unpaired *t*-test. A *P*-value < 0.05 was considered statistically significant.

## 3 Results

### 3.1 Identification and functional analysis of differentially expressed circRNAs and mRNAs in DVT

**Figure 1:**
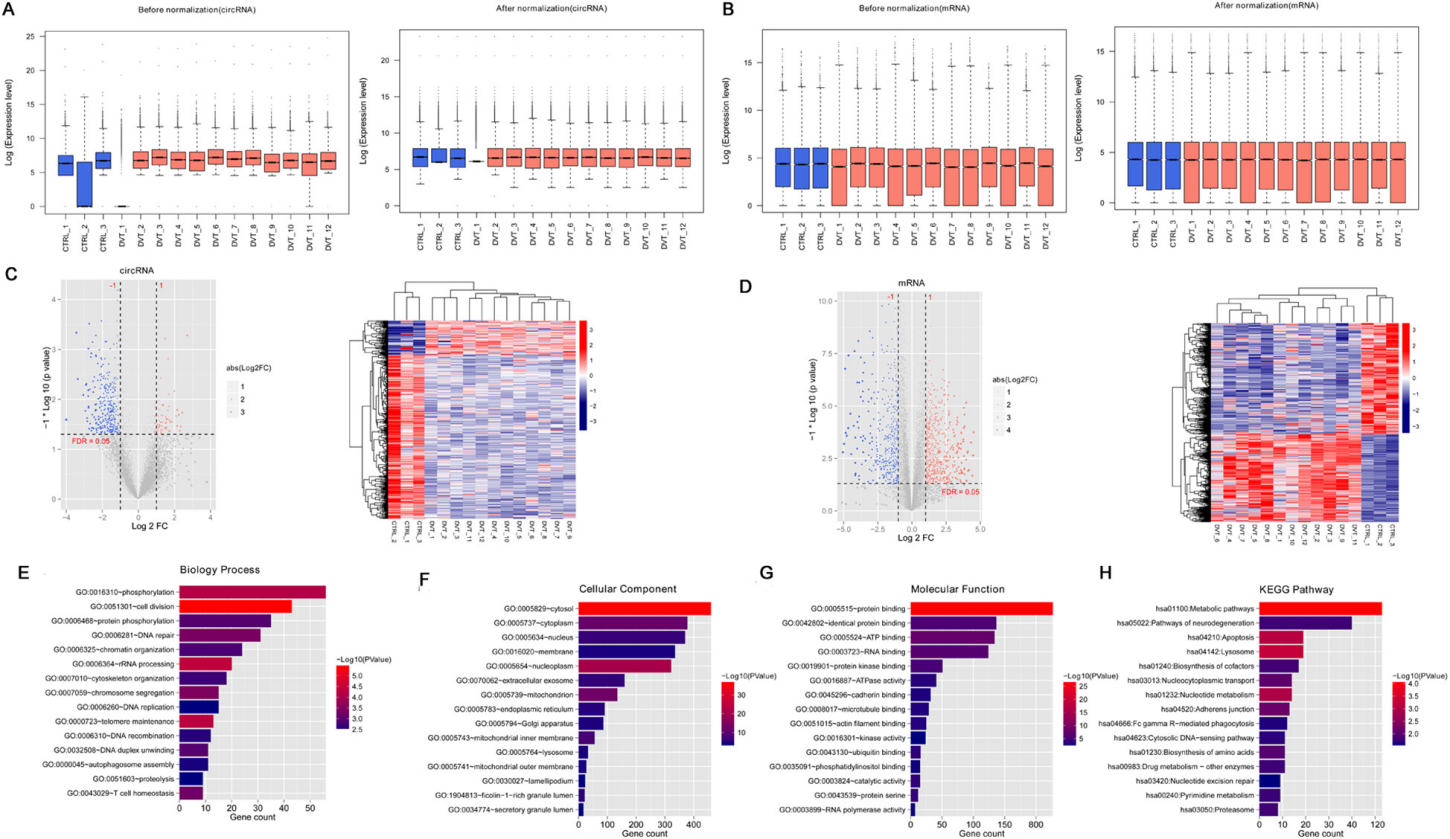
A total of 421 circRNAs and 1082 mRNAs were screened in the deep vein thrombosis (DVT) and control groups. **(A)** Box plot of expression level before and after circRNA standardization; **(B)** box plot of expression level before and after mRNA standardization; **(C)** the expression data of circRNAs showed in a volcano map and heatmap; **(D)** mRNA expression data shown in a volcano map and heatmap. In the volcano map, the horizontal axis is log2FoldChange and the vertical axis is –log10 (*p*-value). The red nodes represent upregulated genes, the blue nodes represent downregulated genes, and the gray nodes represent non-significant differentially expressed genes. **(E)** Top 15 biology processes enriched by 1082 mRNAs; **(F)** top 15 component cellular enriched by 1082 mRNAs; **(G)** top 15 molecular functions enriched by 1082 mRNAs; **(H)** top 15 KEGG pathways enriched by 1082 mRNAs.

To identify differentially expressed circRNAs and mRNAs, data standardization was first performed for circRNAs (Figure 1A) and mRNAs (Figure 1B). After standardization, the expression levels of circRNAs and mRNAs were consistent within the same group (Figures 1A,B). Differential expression analysis using the limma package identified 421 differentially expressed circRNAs (348 downregulated and 73 upregulated circRNAs) (Figure 1C) and 1,082 differentially expressed mRNAs (605 downregulated and 477 upregulated) (Figure 1D) in the DVT group. Hierarchical clustering heatmaps demonstrated that the identified differentially expressed circRNAs and mRNAs could clearly distinguish DVT samples from control samples (Figures 1C,D).

Functional enrichment analysis of the differentially expressed mRNAs revealed significant involvement in 65 BP terms (Figure 1F), including “GO:0051301 ∼ cell division,” “GO:0006364 ∼ rRNA processing,” “GO:0000723 ∼ telomere maintenance”; 63 CC terms (Figure 1F), including “GO:0005829 ∼ cytosol,” “GO:0005654 ∼ nucleoplasm,” “GO:0005739 ∼ mitochondrion”; 41 MF terms (Figure 1G), including “GO:0005515 ∼ protein binding,” “GO:0005524 ∼ ATP binding,” and “GO:0003723 ∼ RNA binding”; and 28 KEGG pathways (Figure 1H), including “hsa01100: Metabolic pathways,” “hsa04142: Lysosome,” and “hsa04210: Apoptosis.”

### 3.2 Determination and functional analysis of the DVT-related circRNAs and mRNAs using WGCNA

**Figure 2:**
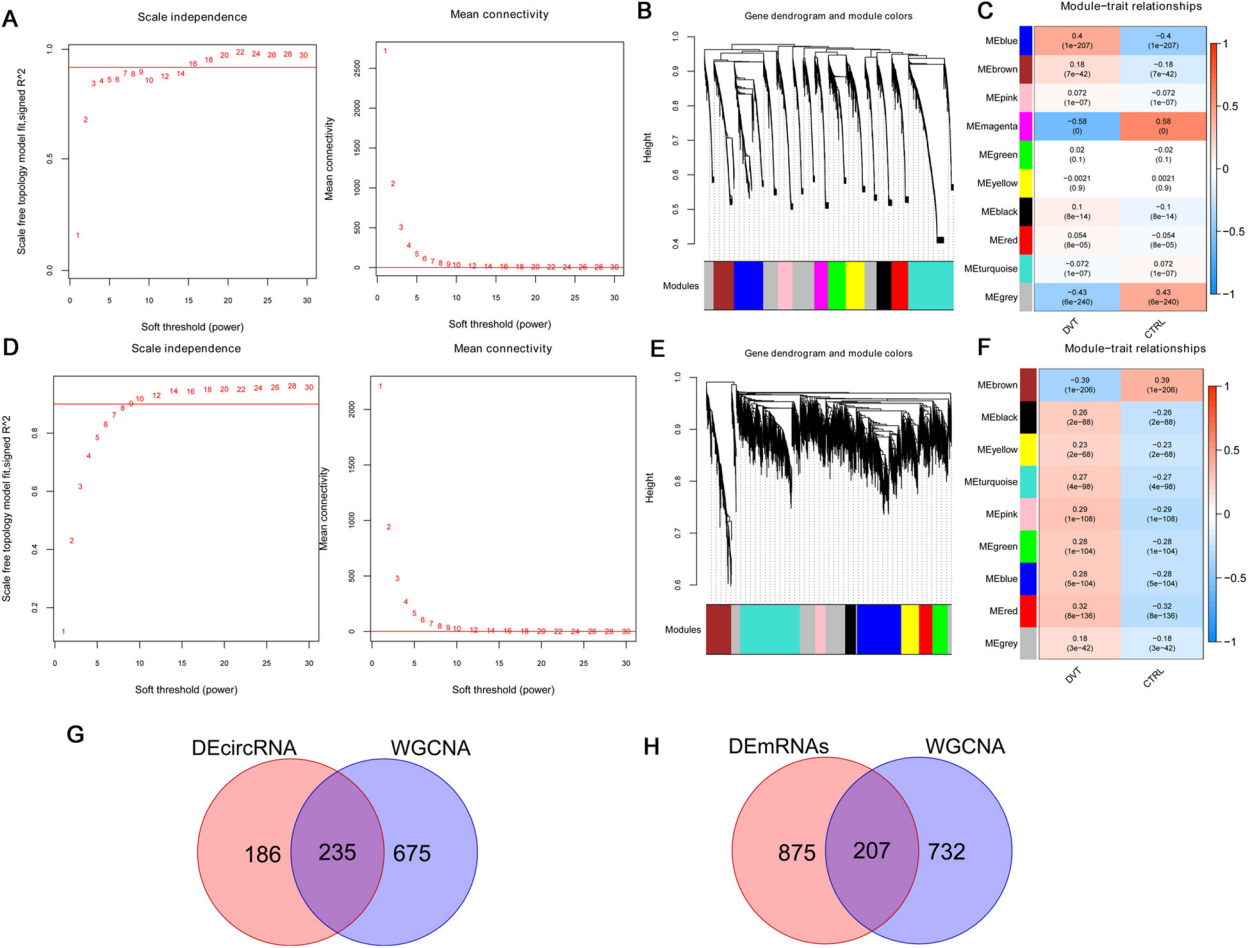
Among differentially expressed genes, 207 DVT-related mRNAs and 235 DVT-related circRNAs were selected by WGCNA. For mRNAs: **(A)** right: selection diagram of the adjacency matrix weight parameter power. The horizontal axis represents the weight parameter power and the vertical axis represents the square of the correlation coefficients between log (*k*) and log (*p* (*k*)) in the corresponding network. The red line represents the standard line where the square value of the correlation coefficient reaches 0.9. Left: a schematic diagram of the average connectivity of genes under different power parameters. The red line represents the value of the average connectivity of network nodes (1) under the weight parameter power of the adjacency matrix in the left figure; **(B)** module partitioning tree diagram with each color representing a different module; **(C)** module-trait correlation heatmap. For circRNAs: **(D)** right: selection diagram of the adjacency matrix weight parameter power. The horizontal axis represents the weight parameter power and the vertical axis represents the square of the correlation coefficients between log (*k*) and log (*p* (*k*)) in the corresponding network. The red line represents the standard line where the square value of the correlation coefficient reaches 0.9. Left: a schematic diagram of the average connectivity of genes under different power parameters. The red line represents the value of the average connectivity of network nodes (1) under the weight parameter power of the adjacency matrix in the left figure; **(E)** module partitioning tree diagram with each color representing different module; **(F)** module-trait correlation heatmap; **(G)** Venn diagram of circRNA; **(H)** Venn diagram of mRNA.

For mRNAs, when the power was set to 16, the average node connectivity of the constructed co-expression network was 1 (Figure 2A). The minimum number of genes for each module was set to 200 and the cutHeight to 0.995, resulting in the identification of 10 modules (Figure 2B). Furthermore, four modules (blue, magenta, brown, and red, involving 939 mRNAs) showed an absolute correlation value higher than 0.3 and were retained (Figure 2C).

For circRNAs, when the power was set to 9, the average node connectivity of the constructed co-expression network was 1 (Figure 2D). The minimum number of genes for each module was set to 200 and the cutHeight to 0.995, resulting in the identification of nine modules (Figure 2E). Of these, three modules (blue, brown, and red, involving 910 circRNAs) with an absolute correlation value higher than 0.3 were retained (Figure 2F).

Next, the selected DVT-related circRNAs and mRNAs were compared with the aforementioned differentially expressed circRNAs and mRNAs, resulting in 235 overlapping circRNAs (Figure 2G) and 207 overlapping mRNAs (Figure 2H), which were used for subsequent analysis.

**Figure 3:**
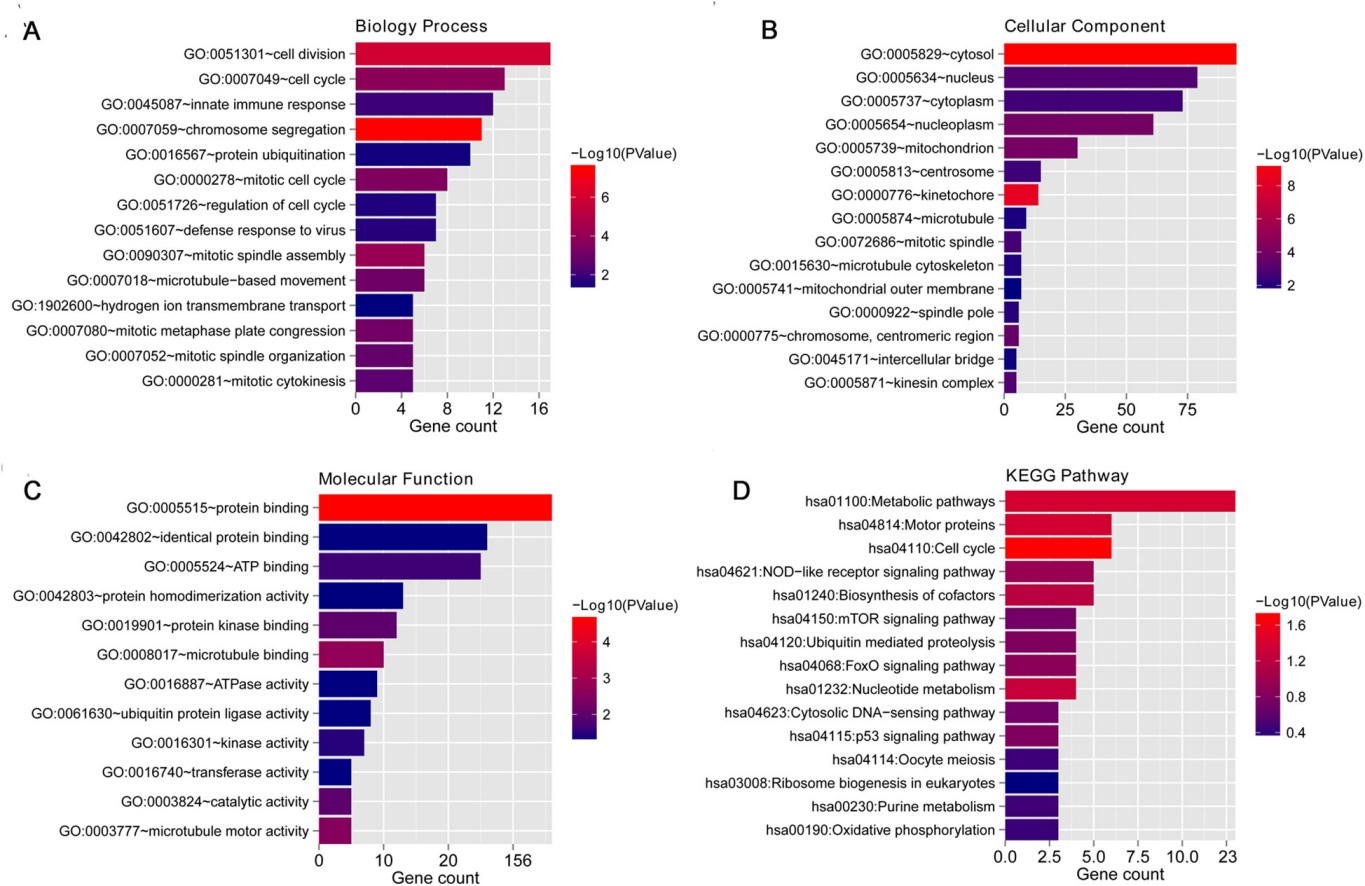
Functional enrichment analysis of the 207 overlapping mRNAs. **(A)** Top 15 biology processes enriched by overlapping mRNAs; **(F)** top 15 component cellular enriched by overlapping mRNAs; **(G)** top 15 molecular functions enriched by overlapping mRNAs; **(H)** top 15 KEGG pathways enriched by overlapping mRNAs.

Functional analysis of the 207 overlapping mRNAs showed significant enrichment in 14 BP terms (Figure 3A), including “GO:0007059 ∼ chromosome segregation,” “GO:0051301 ∼ cell division,” and “GO:0090307 ∼ mitotic spindle assembly”; 20 CC terms (Figure 3B), including “GO:0005829 ∼ cytosol,” “GO:0000776 ∼ kinetochore,” and “GO:0005739 ∼ mitochondrion”; 12 MF terms (Figure 3C), including “GO:0005515 ∼ protein binding,” “GO:0008017 ∼ microtubule binding,” and “GO:0003777 ∼ microtubule motor activity”; and 16 KEGG pathways (Figure 3D), including “hsa04110: Cell cycle,” “hsa01100: Metabolic pathways,” “hsa04814: Motor proteins,” “hsa04621: NOD-like receptor signaling pathway,” “hsa04150: mTOR signaling,” “hsa04068: FoxO signaling pathway,” and “hsa04115: p53 signaling pathway.”

### 3.3 Construction of a PPI network and screen of the hub genes

**Figure 4:**
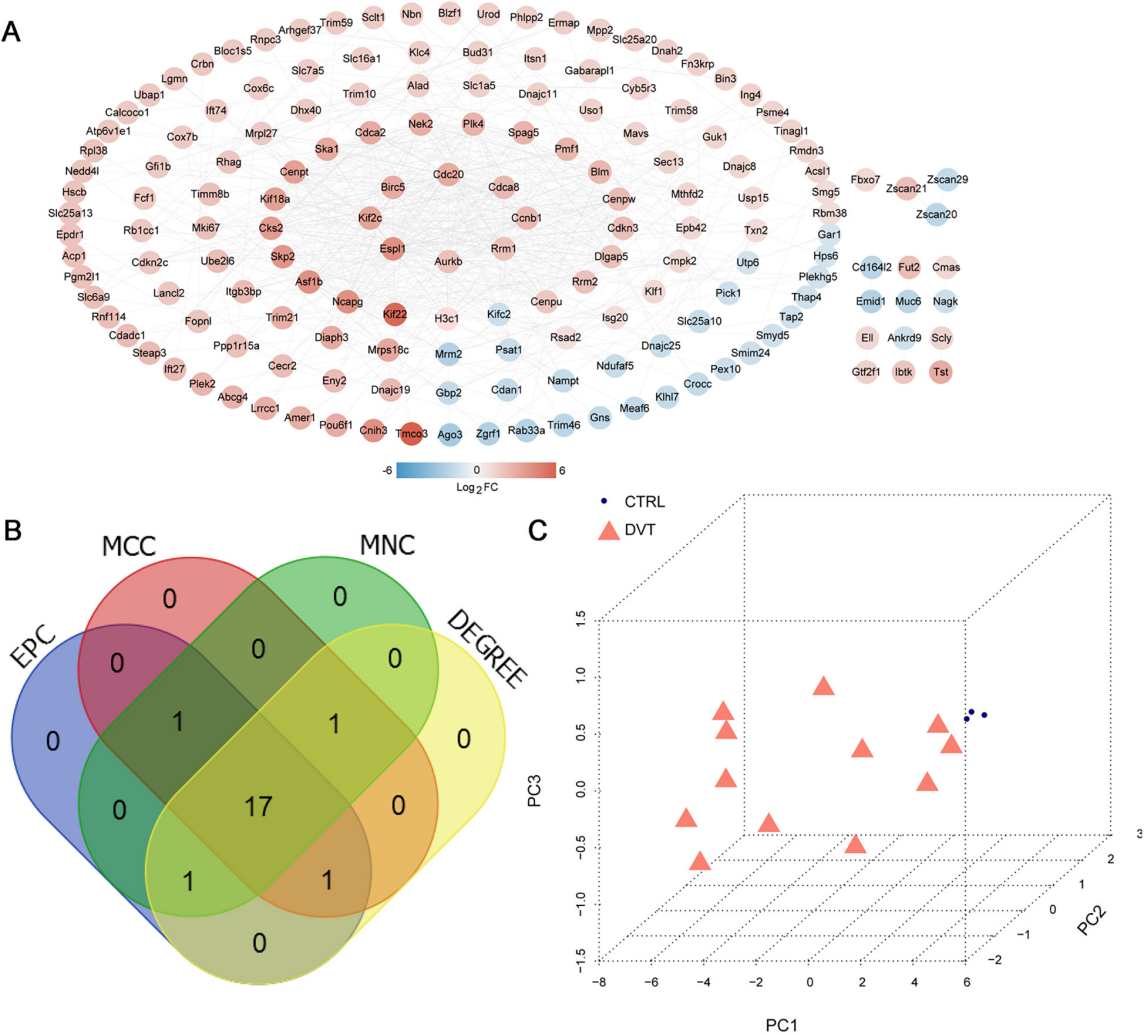
PPI network construction and hub genes selection. **(A)** PPI network based on overlapping mRNAs; color indicates the degree of significant difference; **(B)** Venn diagram for selecting hub genes based on four algorithms, including MCC, MNC, EPC, and DEGREE. **(C)** Principal Component Analysis (PCA) based on 17 hub genes.

The 207 overlapping DVT-related mRNAs were submitted for the construction of a PPI network. From this PPI network, 205 overlapping mRNAs and 573 protein pairs were obtained (Figure 4A). To identify key hub genes, four algorithms—MCC, MNC, EPC, and DEGREE—were applied. After comparing these results, 17 overlapping hub genes were identified: *Birc5*, *Plk4*, *Dlgap5*, *Spag5*, *Cdca2*, *Ccnb1*, *Cdca8*, *Kif18a*, *Kif2c*, *Espl1*, *Cenpu*, *Cdc20*, *Ncapg*, *Asf1b*, *Nek2*, *Aurkb*, and *Cenpw* (Figure 4B). The PCA analysis showed that these hub genes could effectively distinguish between the two groups (Figure 4C).

### 3.4 Establishment of a regulatory circRNA–miRNA–hub gene network

**Figure 5:**
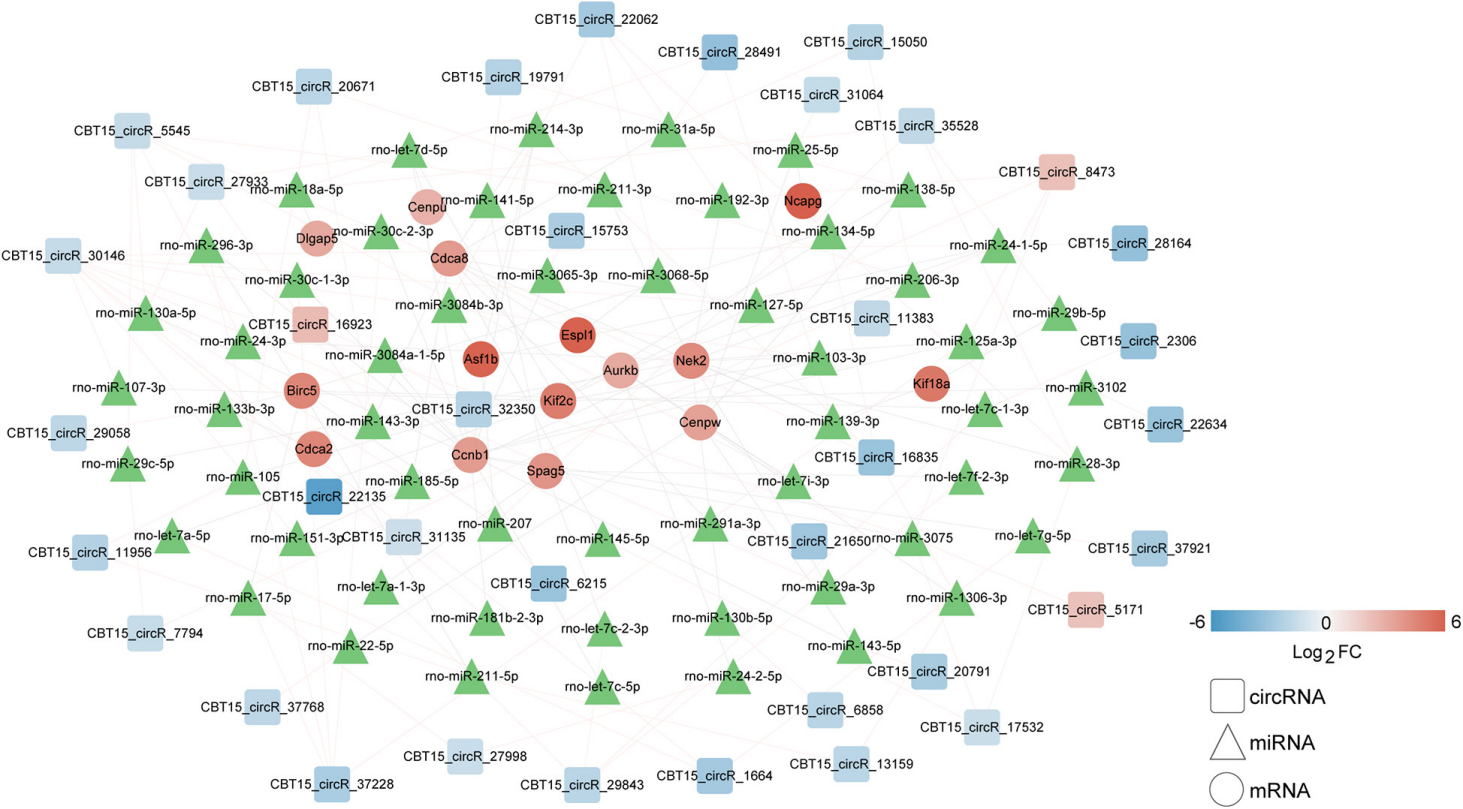
Establishment of a circRNA–miRNA–hub gene network based on expression levels of 17 hub genes. Squares, triangulars, and circles represent circRNAs, miRNAs, and hub genes, respectively. The blue represents downregulation and the red represents upregulation. The gradient intensity of the color bands corresponds to the magnitude of log_2_FC values. miRNAs, which were derived from public databases without available expression quantitation, are uniformly displayed in green to distinguish them from experimentally quantified molecules.

Based on the miRbase database, 227 circRNA–miRNA interaction pairs were identified. In addition, 84 miRNA–hub genes pairs were obtained based on the miRWalk 3.0. Based on the relationship between the circRNA–miRNA and miRNA–hub gene pairs, a regulatory circRNA–miRNA–hub gene network for DVT-related hub genes was constructed, including *CBT15_circR_28491-rno-miR-139-3p-Kif18a/Cdca8/Nek2* axis (Figure 5). The 17 hub genes within the network revealed significant involvement in three KEGG pathways: “rno04110: Cell cycle,” “rno04114: Oocyte meiosis,” and “rno04814: Motor proteins” (Table 2).

**Table 2:** KEGG pathways enriched by 17 hub genes.

Term	Count	*P* value	Genes
rno04110: Cell cycle	3	0.002668	CCNB1, ESPL1, AURKB
rno04114: Oocyte meiosis	2	0.046157	CCNB1, ESPL1
rno04814: Motor proteins	2	0.04983	KIF18A, KIF2C

### 3.5 Correlation analysis of hub genes and immune characteristics

**Figure 6:**
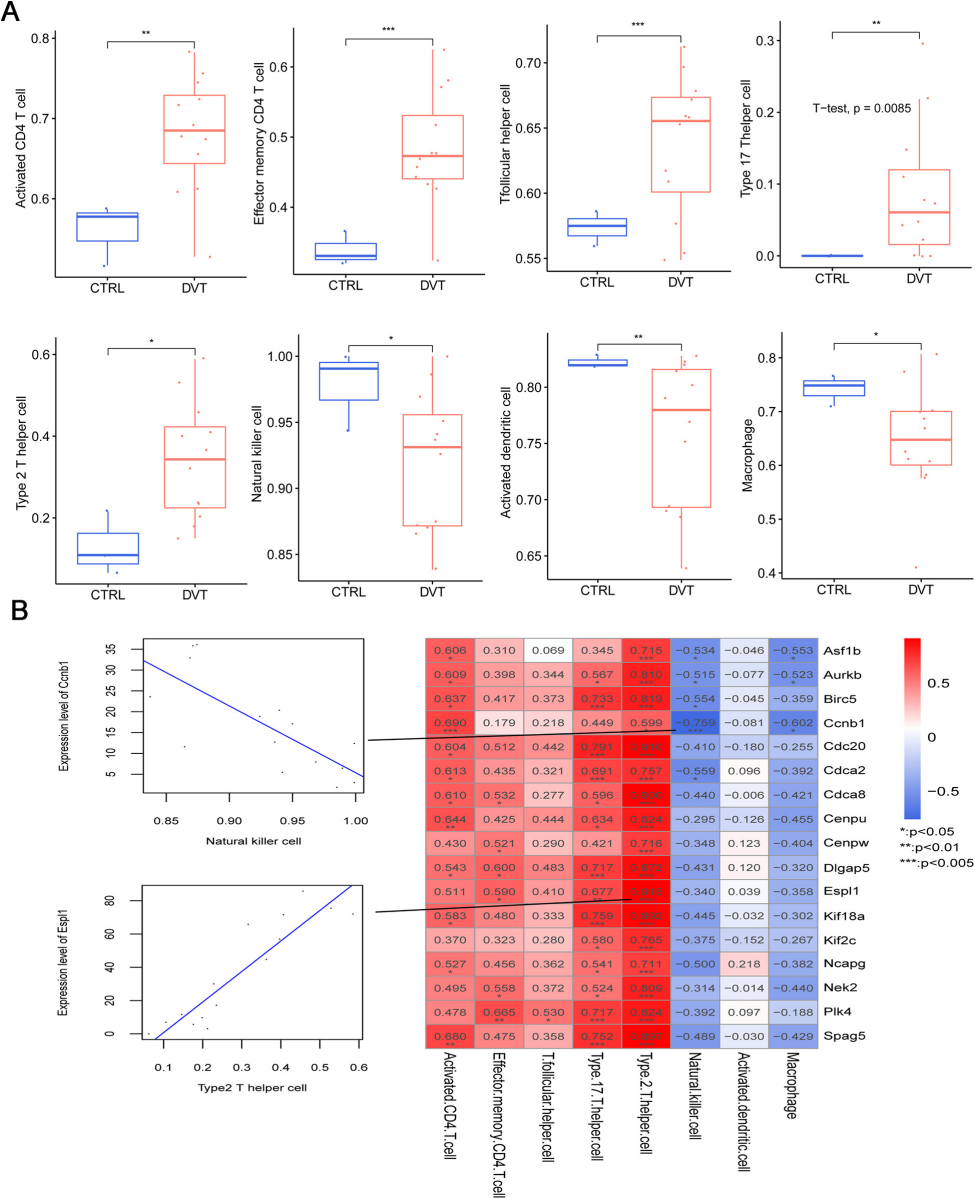
Relationships between the immune cells and 17 hub genes in deep vein thrombosis (DVT). **(A)** The distribution of eight types of immune cells with significantly different proportions in DVT and control groups; **(B)** the correlation between the infiltration levels of the eight immune cells with significant differences and expression levels of 17 hub genes in DVT.

Further, we evaluated the correlation between immune infiltration and the identified hub genes. Analysis revealed eight immune cell types with differential infiltration levels between DVT and control groups, including activated CD4 T cells, effector memory CD4 T cells, T follicular helper cells, type 17 T helper cells, type 2 T helper cells, natural killer cells, activated dendritic cells, and macrophages (Figure 6A). Correlation heatmap analysis showed that type 2 T helper cells were significantly and positively correlated with all 17 hub genes. In contrast, natural killer cells showed significant negative correlations with *Ccnb1*, *Asf1b*, *Aurkb*, *Birc5*, and *Cdca2* (Figure 6B). Moreover, macrophages were negatively associated with *Ccnb1*, *Asf1b*, and *Aurkb* (Figure 6B).

### 3.6 RT-qPCR verification of the selected crucial circRNAs and hub genes

**Figure 7:**
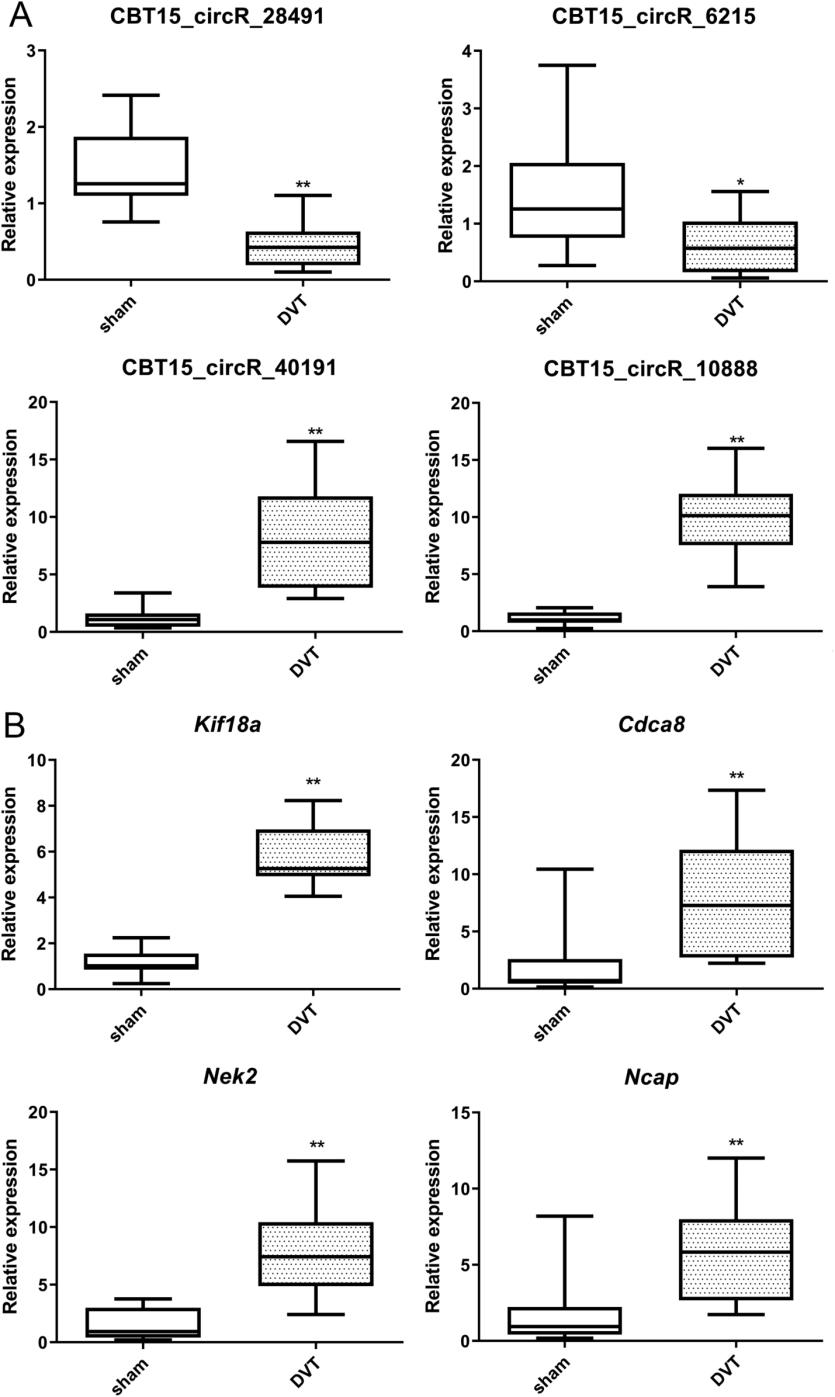
RT-qPCR verification of the selected crucial circRNAs and hub genes. **(A)** The expression levels of CBT15_circR_28491, CBT15_circR_6215, CBT15_circR_10888, and CBT15_circR_40191 in DVT and sham rats. **(B)** The expression levels of *Kif18a*, *Cdca8*, *Nek2*, and *Ncapg* in DVT and sham rats. *p < 0.05, **p < 0.01, vs. sham.

Finally, the expression levels of key circRNAs (CBT15_circR_28491, CBT15_circR_6215, CBT15_circR_10888, and CBT15_circR_40191) and important hub genes (*Kif18a*, *Cdca8*, *Nek2*, and *Ncapg*) were validated in the sham and DVT groups using RT-qPCR. Compared to the sham group, the expression levels of CBT15_circR_28491 and CBT15_circR_6215 were significantly lower, whereas the expression levels of CBT15_circR_10888 and CBT15_circR_40191 were evidently higher in the DVT group (*p* < 0.05) (Figure 7A). As for the hub genes, the expression levels of *Kif18a*, *Cdca8*, *Nek2*, and *Ncapg* were significantly upregulated in DVT rats compared with sham rats (*p* < 0.05) (Figure 7B). Overall, the RT-qPCR results were consistent with the bioinformatic analyses for circRNAs and mRNAs, which were both 100% and 100%, showing the high reliability of our bioinformatic analyses.

## 4 Discussion

DVT, a serious form of venous thromboembolism (VTE), can lead to high mortality and morbidity. Clinically, causative factors for DVT include venous stasis and surgical trauma. Pathologically, thrombosis is caused by activation of exogenous coagulation pathways and venous endothelial cells. In the present study, we identified 235 DVT-related circRNAs and 207 DVT-related mRNAs, which were significantly enriched in signaling pathways such as NOD-like receptor, mTOR, FoxO, p53, and cell cycle. The NOD-like receptor (NLR) protein family, a group of pattern recognition receptors, is known to mediate the initial innate immune response to cellular stress and damage (32). A previous study showed that resveratrol can improve DVT-induced inflammation by inhibiting the HIF-1α/NLRP3 pathway (33). The mTOR pathway integrates diverse environmental cues, such as growth factor signaling and nutrient status, to govern eukaryotic cell growth. By regulating critical cellular processes, including protein synthesis and autophagy, mTOR controls biomass accumulation and metabolism, thereby playing a central role in maintaining cellular and physiological homeostasis (34). Qiao et al. (35) reported that berberine could relieve the functions of endothelial progenitor cells and wound healing *in vivo* via miR-21-3p/RRAGB (a mTOR-related gene), thereby having potential for venous ulcers of the lower extremities. The FoxO signaling pathway determines the fate of cells and is involved in both mitochondrial-dependent and -independent processes during apoptosis, triggering the expression of death receptor ligands [such as Fas ligands and tumor necrosis factor (TNF) apoptotic ligands] (36). The p53 transcription factor is a hallmark of nearly every type of tumor and is closely related to protecting cellular DNA integrity and regulating cell development, aging, and differentiation (37). In a study by Song et al. (38), a novel compound Zn(II)-based coordination polymer showed positive effects on DVT by reducing p-ERK2 and p53 expression. Furthermore, the cell cycle is a series of events that occur in a cell that drive cell division and produces two new daughter cells, which is closely associated with disease progression (39). These findings, together with our results, support the hypothesis that the NOD-like receptor, mTOR, FoxO, p53, and cell cycle pathways may be closely linked to DVT pathogenesis and development. Nevertheless, further research is needed to examine the intricate roles and detailed mechanisms of these pathways in the pathogenesis of DVT.

In addition, we identified 17 hub genes associated with DVT development. RT-qPCR results showed that CBT15_circR_28491 and CBT15_circR_6215 were significantly downregulated, whereas CBT15_circR_10888, CBT15_circR_40191, *Kif18a*, *Cdca8*, *Nek2*, and *Ncapg* were evidently upregulated in the DVT group. Moreover, in our established circRNA–miRNA–hub gene regulatory network, the CBT15_circR_28491-rno-miR-139-3p*-Kif18a/Cdca8/Nek2* axis may play a critical role in DVT development. DVT, a type of endothelial injury, is associated with circulating endothelial progenitor cells and mature circulating endothelial cells (CECs). After thrombus induction, CEC counts increase significantly and remain elevated in DVT patients compared to healthy individuals (40). Therefore, genes involved in cell activity, including cell cycle regulation and cell proliferation, may be important targets for DVT. Previous data have shown that proliferation, invasion, and migration of various kinds of tumor cells were related to miRNA-139-3p (41). *KIF18A*, a member of the kinesin family, is essential for regulating chromosome arrangement during mitosis (38, 39). The gene is a motile microtubule depolymerase essential for chromosome congression, is widely recognized as an oncogene, and is implicated in the progression of various tumors (42). MiRNA-139-3p can bind to *KIF18B* mRNA 3′UTR. In urothelial carcinoma of the bladder, the axis of miR-139-3p/KIF18B/Wnt/β-catenin was demonstrated to be an effective therapeutic target by inhibiting the malignant progression of the tumor (43). Ke et al. reported *miR-139-3p* could suppress gastric cancer progression by targeting *KIF18A* (44). *Cdca8*, a critical regulator of mitosis (45), has been linked to increased growth of tumor cells (46). *Nek2*, a mitotic Ser/Thr kinase, plays a key role in controlling cell proliferation, growth, and differentiation (47). *Ncapg*, overexpressed in many cancers, can serve as a new diagnostic and therapeutic target and is also a very promising prognostic marker (48). To date, there is no research on the roles of CBT15_circR_28491, CBT15_circR_6215, CBT15_circR_10888, and CBT15_circR_40191 on DVT. A study by Wang et al. reported that hsa_circRNA_092488 was higher in DVT and may exacerbate the progression of DVT via the NLRP3/NF-κB signaling pathway (49). Taken together, the identified circRNAs and hub genes, as well as the CBT15_circR_28491-miR-139-3p*-Kif18a/Cdca8/Nek2* axis, may be closely linked to the thrombotic risk associated with neoplasia and play essential roles in the progression of DVT. However, further *in vitro* and *in vivo* studies are necessary to examine the underlying mechanisms of the CBT15_circR_28491-miR-139-3p-*Kif18a*/*Cdca8*/*Nek2* pathway in DVT.

In addition, our study identified differential infiltration levels of eight immune cell types in DVT, with type 2 T helper cells showing a significant positive correlation with all the 17 hub genes. Previous research by Luo et al. showed that, compared with stable plaques, unstable plaques exhibited highly negative regulators of coagulation expressed by T helper cells, including PLAUR, PLAU, PLG, and PROCR (50). Meanwhile, activated human T helper cells were found to demonstrate higher fibrinolytic activity than their non-activated counterparts (50). Further evidence suggests that T helper cell activity can act either as a risk or protective factor in various cardiovascular diseases, such as myocardial infarction, coronary heart disease, and stroke (51, 52). Our data also showed that T helper cells were positively associated with all 17 hub genes. Therefore, a close link is suggested between T helper cell activation and the development of DVT.

In conclusion, this study provides valuable information about circRNA and mRNA profiles in DVT. It highlights the valuable roles of CBT15_circR_28491-miR-139-3p-*Kif18a*/*Cdca8*/*Nek2* and T helper cells in DVT. These findings deepen our understanding of the pathogenesis of DVT and provide novel therapeutic targets (CBT15_circR_28491-miR-139-3p-*Kif18a*/*Cdca8*/*Nek2*) for DVT management.

## Data Availability

The datasets presented in this study can be found in online repositories. The names of the repository/repositories and accession number(s) can be found below: NCBI GEO (http://www.ncbi.nlm.nih.gov/geo/), GSE148333.
